# The effect of SNP rs400827589 in exon 2 of the *MTNR1B* gene on reproductive seasonality and litter size in sheep

**DOI:** 10.1002/vms3.280

**Published:** 2020-05-06

**Authors:** Xiaoyun He, Zhuangbiao Zhang, Mingxing Chu

**Affiliations:** ^1^ Key Laboratory of Animal Genetics and Breeding and Reproduction of Ministry of Agriculture Institute of Animal Science Chinese Academy of Agricultural Sciences Beijing China

**Keywords:** litter size, MTNR1B, reproductive seasonality, sheep, SNP

## Abstract

In mammals, the melatonin receptor gene has been widely studied since it has a great influence on reproductive traits. However, little is known about the association between polymorphism of the coding region of the *MTNR1B* gene and year‐round oestrus or the litter size in Small Tail Han sheep. To better understand the effects of single nucleotide polymorphism (SNP) rs400827589 in *MTNR1B*, a population polymorphism analysis was conducted using genotyping data in 45 sheep breeds around the world. The results indicated that TT was the dominant genotype in all sheep breeds. The associations of this SNP with reproductive seasonality and litter size in Small Tail Han sheep showed rs400827589 was correlated with fecundity as assessed by reproductive seasonality and litter size (*p* < .05). Bioinformatics analysis indicated the change in amino acid from Ile to Leu may affect the function of the MTNR1B protein by impacting the secondary and tertiary protein structures. The present results demonstrate that rs400827589 could be used in the marker‐assisted selection of the litter size in Small Tail Han sheep.

## Graphical abstract

rs400827589 was correlated with fecundity as assessed by reproductive seasonality and litter size (*p* < .05). Bioinformatics analysis indicated the change of amino acid from Ile to Leu may affect the function of the MTNR1B protein by impacting the secondary and tertiary protein structures

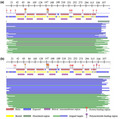

## 1 INTRODUCTION

Melatonin is a vital hormone that is secreted mainly by the pineal gland, and has a significant effect on several physiological functions including circadian rhythm and reproduction through binding its special receptors in mammals (Calvo et al., 2018; Chu et al., 2007; Chu, Ji, & Chen, 2003; Hardeland, 2017; Lei, Di, Liu, & Chu, 2015; Li et al., 2013; Ramírez et al., 2009) and birds (Alsiddig et al., 2016; Amit Kumar & Vinod, 2014; Feng et al., 2018; Rajesh & Chandana, 2011; Zhao et al., 2014). There are three melatonin receptor subtypes, melatonin receptor 1A (MTNR1A, MT1), melatonin receptor 1B (MTNR1B, MT2) and melatonin receptor 1C (MTNR1C), which belong to the superfamily of G protein‐coupled receptors (Dubocovich & Markowska, 2005; Reiter, 1980). Many investigators showed that melatonin is involved in folliculogenesis, follicle selection, oocyte maturation as well as regulating the granulosa cells secretion (Dubocovich & Markowska, 2005; Tamura et al., 2009; Wang et al., 2017; Wang, Liu, Ahmad, et al., 2012; Wang, Liu, Wu, et al., 2012).

To date, numerous studies have explored the relationship between polymorphism of *MTNRs* and litter size or reproductive seasonality traits in different mammals (Calvo et al., 2018; Chu, Cheng, Liu, Fang, & Ye, 2006, 2008; Chu et al., 2007; Gunwant et al., 2018; Hua, 2006; Liu, Wang, Zhou, Pang, & Wang, 2018; Notter, Cockett, & Hadfield, 2003; Pelletier et al., 2000; Wang, Liu, Ahmad, et al., 2012; Wang, Liu, Wu, et al., 2012). However, there were few studies about the polymorphism of *MTNRIB*. Interestingly, some studies in birds found that melatonin receptor subtypes were identified in ovaries (He et al., 2014; Sundaresan et al., 2009; Wang et al., 2008), which indicated that melatonin may directly affect ovarian function through activating of its receptors. For example, the expression levels of MTNR1A, MTNR1B and MTNR1C initially increased and later decreased during the follicular development cycle in geese, suggested that melatonin receptors participated in activating small follicles to develop into subsequent higher hierarchical follicles (He et al., 2014). Besides, in a recent study, it was demonstrated that melatonin could directly modulate bovine ovarian function through MTNR1B, melatonin and MTNR1B were involved in the BCL2 family and CASP3‐dependent apoptotic pathways in bovine granulosa cells (Liu et al., 2018; Wang, Liu, Ahmad, et al., 2012; Wang, Liu, Wu, et al., 2012). In addition, authors also found rs10830963 and rs10830962 SNPs in *MTNR1B* gene were associated with female polycystic ovary syndrome (PCOS) (Li, Shi, You, Wang, & Chen, 2011; Yang, Yang, & Cheng, 2016), this result suggests that SNPs in *MTNR1B* gene may affect integrity of ovarian function to regulate reproductive activity.

The prolificacy and year‐round oestrus of sheep are crucial economic traits. Small Tail Han sheep, a local sheep breed in China, is characterized by high productivity and year‐round oestrus (Liu, Jiang, & Du, 2003), and the breed has been considered as a good source of ovine reproductive genes in China. In the early studies, studies reported that the relationship between polymorphism of *MTNR1A* and litter size and reproductive seasonality traits in different breeds of ruminants (Gunwant et al., 2018), such as sheep (Calvo et al., 2018; Chu et al., 2006, 2008; Mateescu, Lunsford, & Thonney, 2009; Notter et al., 2003; Pelletier et al., 2000; Posbergh, Murphy, & Thonney, 2017), goat (Abdolahi, Shokrollahi, Saadati, & Morammazi, 2019; Chu et al., 2007; Hua, 2006) and water buffalo (Gunwant et al., 2018). However, there are few reports about *MTNR1B*. In our early study on variation of the genome in 89 sheep, we found a significant single nucleotide polymorphism rs400827589 in the re‐sequencing data (Pan et al., 2018), and several other significant SNPs from which have been published in our early reports (He, Zhang, Liu, & Chu, 2019; Zhou et al., 2018). Therefore, in order to better understand the function of this mutation in *MTNR1B*, we explored the polymorphic distribution using a large population and then investigated the association with litter size and reproductive seasonality in sheep.

## 2 MATERIALS AND METHODS

### 2.1 Ethics statement and sample preparation

All animals used in this study were approved by the Science Research Department (in charge of animal welfare issues) of the Institute of Animal Science, Chinese Academy of Agricultural Sciences (IAS‐CAAS). In addition, there was ethics approval by the animal ethics committee of IAS‐CAAS (No. IAS2018‐3).

Jugular vein blood samples were collected from 737 ewes in six sheep breeds for DNA isolation using the phenol‐chloroform method. The information of sheep breeds included in the study was shown in Table 1.

**TABLE 1:** Information of six sheep breeds selected for genotyping

Breed	Number	Type	District
Small Tail Han	380	Multiple lambs and year‐round oestrus	Yuncheng, Shandong Province, China
Hu	101	Multiple lambs and year‐round oestrus	Xuzhou, Jiangsu Province, China
Cele black	52	Multiple lambs and year‐round oestrus	Cele, Hetian, Xinjiang Uygur Autonomous Region, China
Prairie Tibetan	161	SINGLE birth and seasonal oestrus	Dangxiong, Tibet Autonomous Region, China
Sunite	21	SINGLE birth and seasonal oestrus	Wulatezhongqi, Bayannaoer, Inner Mongolia Autonomous Region, China
Tan	22	Single birth and seasonal oestrus	Yanchi, Ningxia Hui Autonomous Region, China

### 2.2 Genotyping the candidate SNP in *MTNR1B* gene

After DNA extraction, the primers used for genotyping the rs400827589 were designed using the MassARRAY Assay Design v. 3.1 according to sheep *MTNR1B* sequences available in the *Ensembl* (Accession No. ENSOARG00000002933), the primer sequences for genotyping were 5′‐TGG ATG AAC AAC CCC TCT GGG ATC CG‐3′ (Forward), 5′‐ACG TTG GAT GTT GTG ATC TTC GCC ATC TGC‐3′ (Reverse) and 5′‐TTG TGA GCC ACT TCT TCG GGG TCA/A3′ (Extension reaction), which amplify a region of 120bp. The MassARRAY^®^SNP analysis (http://www.sequenom.com) was subsequently applied for genotyping all 737 sheep. The polymerase chain reactions system and temperature were described in detail in a previous study (Zhou et al., 2018).

### 2.3 Statistical analysis

The calculations of allele frequencies and genotype frequencies and the Hardy‐Weinberg equilibrium tests were performed by using Popgene (version 1.31) (Chong, Huang, Liu, Jiang, & Rong, 2018). The association analysis between polymorphisms of the *MTNR1B* gene and the litter size or year‐round oestrus was conducted by using General Linear Model in SAS (v 9.2) (SAS Institute Inc.). *p* values less than .05 were considered to be significant. The model was described in the previous study (Zhang et al., 2019), which was as follows: *y_ijn_* = *μ* + P*_i_* + G*_j_* + I*_PG_* + *e_ijn_*, where *y_ijn_* is the phenotypic value of litter size; *μ* is the population mean; P*_i_* is the fixed effect of the ith parity (*i* = 1, 2, 3); G*_j_* is the fixed effect of the *j*th genotype (*j* = 1, 2, 3); I*_PG_* is the interaction effect of parity and genotype and *e_ijn_* is the random residual.

### 2.4 Bioinformatics analysis

The coding sequences of the *MTNR1B* gene were obtained from NCBI (https://www.ncbi.nlm.nih.gov/nuccore/NM_001130938.1), and amino acid sequences were subsequently obtained from NCBI (https://www.ncbi.nlm.nih.gov/protein/195972821). The transmembrane domains before and after mutation in MTNR1B were predicted using TMHMM (http://www.cbs.dtu.dk/services/TMHMM‐2.0/). Prediction of the secondary structure of MTNR1B and its mutants occurred using Predict Protein (https://www.predictprotein.org/). The MTNR1B protein three‐dimensional (3D) structure in sheep was predicted by Iterative Threading ASSEmbly Refinement (I‐TASSER) (http://zhanglab.ccmb.med.umich.edu/I‐TASSER/), the protein‐ligand binding site prediction was performed by a meta‐server approach (COACH) (http://zhanglab.ccmb.med.umich.edu/COACH/).

## 3 RESULTS

### 3.1 Population polymorphism analysis of polymorphism in the *MTNR1B* gene

In this study, rs400827589 in *MTNR1B* exon 2 was selected for genotyping, the different alleles resulted in amino acid changes. The genotype results in 737 samples with a >95% success rate and samples with successful genotyping were included in the population polymorphism analysis (Table 2). The results indicated that the three genotypes including TT, TG and GG were all detected. All six sheep breeds showed low polymorphism at this SNP. The GG genotype was only found in Small Tail Han and Hu sheep. The Chi‐square test showed that the distribution of SNP was under Hardy‐Weinberg equilibrium (*p* > .05).

**TABLE 2:** Population polymorphism analysis of locus in six sheep breeds

Locus	Breed	Genotype frequency (count)			Allele frequency		PIC	HE	NE	Chi‐square test (*p*‐value)
g.1373884T > G c.826A > C p.Ile276Leu rs400827589	‐	TT	TG	GG	T	G	—	—	—	—
	Small Tail Han	0.72 (274)	0.25 (95)	0.03 (11)	0.85	0.15	0.22	0.26	1.35	0.85
	Hu	0.79 (80)	0.19 (19)	0.02 (2)	0.89	0.11	0.18	0.20	1.25	0.50
	Prairie Tibetan	0.94 (151)	0.06 (10)	0.00 (0)	0.97	0.03	0.05	0.05	1.06	0.72
	Cele black	0.79 (41)	0.21 (11)	0.00 (0)	0.89	0.11	0.17	0.19	1.23	0.39
	Sunite	0.76 (16)	0.24 (5)	0.00 (0)	0.88	0.12	0.19	0.21	1.27	0.54
	Tan	0.77 (17)	0.23 (5)	0.00 (0)	0.89	0.11	0.18	0.20	1.25	0.55

Note

PIC, HE and NE represent polymorphism information content, heterozygosity and effective number of alleles, respectively; *p* > .05 indicates the locus was under Hardy‐Weinberg equilibrium.

A population polymorphism analysis was also conducted in another 39 sheep breeds, the results are listed in Table 3. The genotype and allele frequency information of those breeds were obtained from NextGen Project and International Sheep Genome Consortium (ISGC) (http://asia.ensembl.org/Ovis_aries/Variation/Population?db=core;g=ENSOARG00000002933;r=21:1373657‐1392132;t=ENSOART00000003171;v=rs400827589;vdb=variation;vf=33224018). The three genotypes were found in different sheep breeds from various countries in the world. The distribution of genotypes was similar to native breeds in China, the dominant genotype was TT and a few sheep breeds had the GG genotype.

**TABLE 3:** Population polymorphism analysis of rs400827589 in sheep from NextGen Project and ISGC

Data sources	Sheep (abbreviation)	Allele frequency (count)		Genotype frequency (count)		
		T	G	TT	GT	GG
NextGen Project	Iranian Ovis aries (IROA)	0.875 (35)	0.125 (5)	0.750 (15)	0.250 (5)	—
	Moroccan Ovis aries (MOOA)	0.781 (250)	0.219 (70)	0.625 (100)	0.312 (50)	0.063 (10)
ISGC	ALL of 37	0.781 (700)	0.219 (196)	0.645 (289)	0.272 (122)	0.083 (37)
	Afshari (AFS)	1.000 (4)	—	1.000 (2)	—	—
	African white dorper (AWD)	1.000 (4)	—	1.000 (2)	—	—
	Awassi (AWS)	1.000 (6)	—	1.000 (3)	—	—
	Bangladeshi (BAN)	1.000 (4)	—	1.000 (2)	—	—
	Beni Guil (BEN)	0.917 (11)	0.083 (1)	0.833 (5)	0.167 (1)	—
	Brazilian Creole (BRA)	0.750 (3)	0.250 (1)	0.500 (1)	0.500 (1)	—
	Castellana (CAS)	0.750 (3)	0.250 (1)	0.500 (1)	0.500 (1)	—
	Cheviot (CHE)	0.250 (1)	0.750 (3)	—	0.500 (1)	0.500 (1)
	Chnagthangi (CHN)	1.000 (4)	—	1.000 (2)	—	—
	Churra (CHU)	0.250 (1)	0.750 (3)	—	0.500 (1)	0.500 (1)
	Composite (CMP)	0.761 (143)	0.239 (45)	0.606 (57)	0.309 (29)	0.085 (8)
	Coopworth (CPW)	0.865 (64)	0.135 (10)	0.757 (28)	0.216 (8)	0.027 (1)
	D’man (DMA)	0.712 (37)	0.288 (15)	0.577 (15)	0.269 (7)	0.154 (4)
	Finn sheep (FIN)	1.000 (8)	—	1.000 (4)	—	—
	Garut (GAR)	1.000 (4)	—	1.000 (2)	—	—
	Gulf coast native (GUL)	0.500 (2)	0.500 (2)		1.000 (2)	—
	Karakas (KAR)	0.750 (3)	0.250 (1)	0.500 (1)	0.500 (1)	—
	Merino horned (MEH)	0.375 (3)	0.625 (5)	0.250 (1)	0.250 (1)	0.500 (2)
	Merino polled (MEP)	0.583 (7)	0.417 (5)	0.333 (2)	0.500 (3)	0.167 (1)
	Merino (MER)	0.833 (5)	0.167 (1)	0.667 (2)	0.333 (1)	—
	Morada nova (MOR)	1.000 (4)	—	1.000 (2)	—	—
	Norduz (NOR)	0.750 (3)	0.250 (1)	0.500 (1)	0.500 (1)	—
	Norwegian white sheep (NWS)	1.000 (4)	—	1.000 (2)	—	—
	Ojalada (OJA)	1.000 (4)	—	1.000 (2)	—	—
	Ouled Djellal (OUL)	0.750 (12)	0.250 (4)	0.625 (5)	0.250 (2)	0.125 (1)
	Romney (ROM)	0.702 (66)	0.298 (28)	0.596 (28)	0.213 (10)	0.191 (9)
	Ronderib Afrikaner (RON)	0.500 (2)	0.500 (2)		1.000 (2)	—
	Sakiz (SAK)	1.000 (4)	—	1.000 (2)	—	—
	Salz (SAL)	1.000 (4)	—	1.000 (2)	—	—
	Santa ines (SAN)	1.000 (4)	—	1.000 (2)	—	—
	Sardinian ancestral black (SAR)	0.729 (35)	0.271 (13)	0.583 (14)	0.292 (7)	0.125 (3)
	Sumatran (SUM)	0.750 (3)	0.250 (1)	0.500 (1)	0.500 (1)	
	Swiss white alpine (SWA)	0.500 (4)	0.500 (4)	0.250 (1)	0.500 (2)	0.250 (1)
	Timahdite (TIM)	0.833 (25)	0.167 (5)	0.667 (10)	0.333 (5)	—
	Texel (TXL)	0.850 (17)	0.150 (3)	0.800 (8)	0.100 (1)	0.100 (1)
	Unclassified (UNK)	0.812 (151)	0.188 (35)	0.667 (62)	0.290 (27)	0.043 (4)
	Wiltshire (WIL)	1.000 (4)	—	1.000 (2)	—	—

We classified six breeds into two categories, year‐round oestrus and seasonal oestrus, based on the oestrous characters. The results of the population polymorphism analysis shown in Table 4, indicated that the rs400827589 was significantly different between year‐round oestrous sheep and seasonal oestrous sheep (*p* < .01).

**TABLE 4:** Genotype and allele frequencies of rs400827589 in MTNR1B in sheep with different oestrous characters

Oestrous characters	Genotype frequency			Allele frequency		Chi‐square test (*p*‐value)
—	TT	TG	GG	T	G	—
Year‐round oestrous	0.77 (411)	0.22 (117)	0.01 (5)	0.88	0.12	5.41E−06
Seasonal oestrous	0.83 (169)	0.17 (35)	0.00 (0)	0.91	0.09	

### 3.2 Association of polymorphism of rs400827589 with litter size in Small Tail Han sheep

The association analysis between rs400827589 with litter size in Small Tail Han sheep was performed using data from our previous study (Zhang et al., 2019), the results are shown in Table 5. Whether in the first, second or third parity, individuals with TT and TG genotypes had a larger litter size than those with GG genotype (*p* < .05).

**TABLE 5:** Least squares means and standard errors of litter size in Small Tail Han sheep with different genotypes

Locus	Genotype	Litter size of the first parity	Litter size of the second parity	Litter size of the third parity
rs400827589	TT	2.14 ± 0.07a (248)	2.35 ± 0.08a (232)	2.72 ± 0.12a (93)
	TG	2.18 ± 0.08a (81)	2.44 ± 0.08a (80)	2.95 ± 0.13a (32)
	GG	1.22 ± 0.30b (8)	1.66 ± 0.32b (8)	1.75 ± 0.25b (4)

Note

Numbers in the parentheses next to litter size represent the amount of sheep of each genotype; Different small letters in the same group mean significant difference (*p* < .05).

### 3.3 Bioinformatics analysis of MTNR1B

**FIGURE 1:**
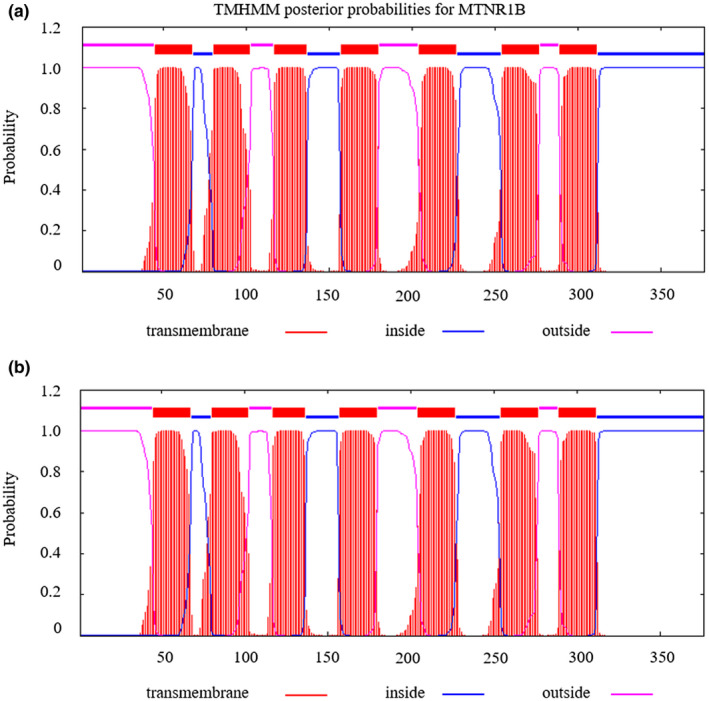
Transmembrane domain prediction of the MTNR1B protein before and after mutation at rs400827589. (a) the transmembrane domain before mutation; (b) the transmembrane domain after mutation

**FIGURE 2:**
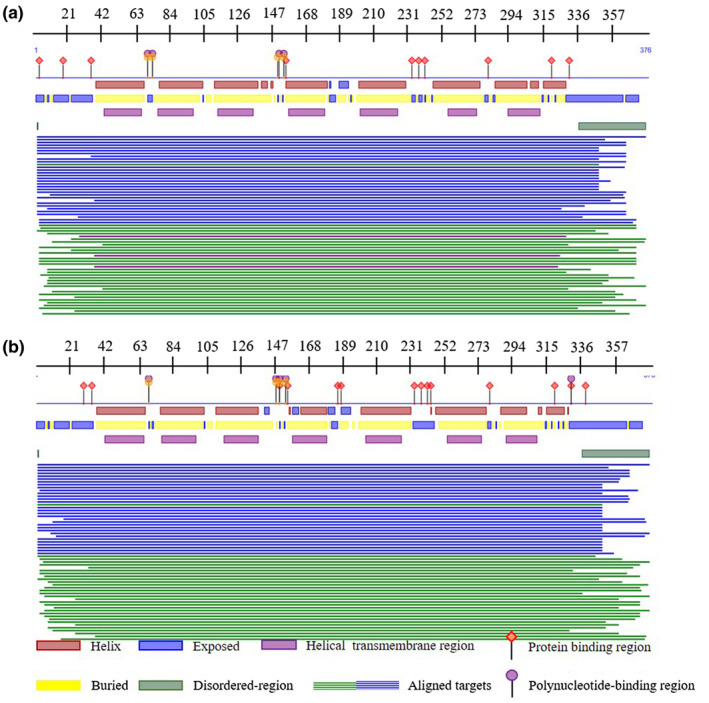
Secondary protein structure for the MTNR1B product before and after the mutation at rs400827589. (a) Secondary protein structure before the mutation; (b) Secondary protein structure after the mutation

**FIGURE 3:**
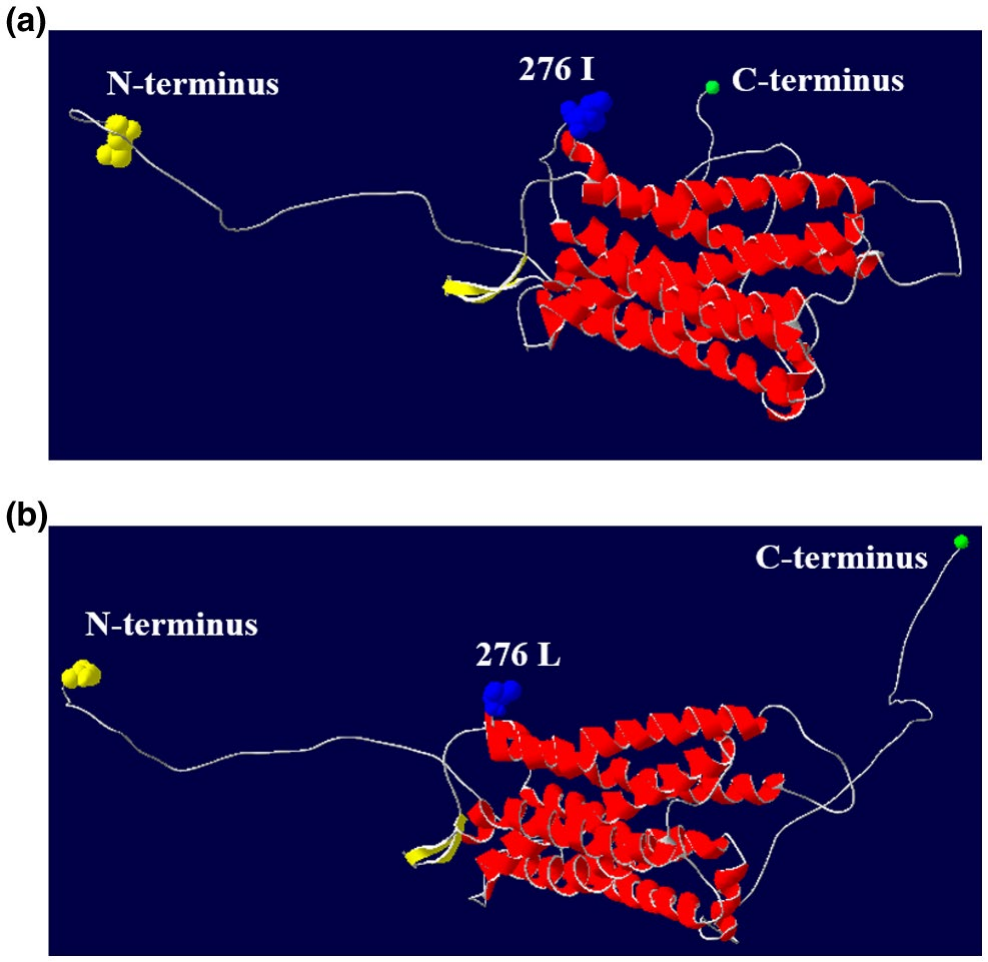
Three‐dimensional structure modeling of the MTNR1B protein in sheep. (a) Three‐dimensional structure before mutation, B, Three‐dimensional structure after mutation

To analyze the changes before and after the mutation of rs400827589, the transmembrane domain was predicted using the amino acid (AA) sequence. The results indicated that there were seven transmembrane domains, a mutation from T to G led to no change in the transmembrane domains (Figure 1). In addition, the protein secondary structure before and after the mutation was predicted using the AA sequence (Figure 2). Near the AA 276 position, there was a protein binding region in both the wild and mutation genotype. Interestingly, a mutation from T to G could lead to the presence of a unique polynucleotide‐binding region in proximity to AA 326 position (Figure 2b). Finally, prediction of the protein tertiary structure was predicted before and after the mutation at rs400827589. The results indicated there were eight α‐helixes and two β‐strands, the locus rs400827589 at the end of the sixth α‐helix (Figure 3). Compared with the wild type, the side chains of amino terminal and carboxyl terminal of the mutation had structural changes, while α‐helix and β‐strand had no change in tertiary structure.

## 4 DISCUSSION

Melatonin is a highly lipophilic circulating hormone. In addition to regulating insulin secretion and glucose levels, it controls circadian rhythms and reproductive processes (Carla Cristina et al., 2013; Jaworek et al., 2007; Sack, Blood, & Lewy, 1992). The function of melatonin is mediated by its receptors MTNR1A or MTNR1B in mammals (Li et al., 2013; Wang, Liu, Ahmad, et al., 2012; Wang, Liu, Wu, et al., 2012). For example, melatonin can regulate the secretion of progesterone by binding to melatonin receptors in granulosa cells, and the gene expression of LH receptor and gonadotropin‐releasing hormone receptor (Li et al., 2011). MTNR1B belongs to the 7‐transmembrane G‐protein coupled receptor superfamily member in human, widely expressed in the hypothalamus, pituitary gland, ovary, uterus, fallopian tubes and testis tissues, most importantly, with the highest expression in the ovaries (Yang et al., 2016).

Recent studies demonstrated that melatonin could directly modulate bovine ovarian function through MTNR1B, melatonin and MTNR1B are involved in the BCL2 family and apoptotic pathways in bovine granulosa cells (Liu et al., 2018; Wang, Liu, Ahmad, et al., 2012; Wang, Liu, Wu, et al., 2012). Besides, polymorphisms of *MTNR1B* gene have a great influence on the egg reproduction trait in avian species, such as chicken (Li et al., 2012; Zhao et al., 2014), duck (Feng et al., 2018) and goose (Alsiddig et al., 2016). However, no data have been reported about polymorphism on *MTNR1B* and its correlation with ovine reproductive seasonality and litter size. In order to better understand the function of SNP rs400827589 SNP, population polymorphism analysis in six sheep breeds showed the three genotypes, including TT, TG and GG, were all detected in Small Tail Han sheep and Hu sheep, but only the TT and TG genotypes were found in the other four breeds. Furthermore, the SNP in all six sheep breeds with a low polymorphism (PIC < 0.25). Compared with the distribution of this locus in sheep breeds around the world (Table 2), we found TT was the dominant genotype, but GG was also detected in many domestic and foreign sheep breeds. These results indicate the SNP mutation might be at an early stage in evolution which provides information for us to further study its functions.

Transmembrane domains are integral components enabling numerous proteins to function (Nadir, Hassan, Daniel, & Walid, 2012; Tarasova, Rice, & Michejda, 1999). Transmembrane domains can also affect reproduction. For example, two mutations of the FSH receptor in the transmembrane domain can cause primary ovarian failure (Bramble et al., 2016). Our early studies in BMP2 found a transmembrane domain change after a mutation in *BMP2* at g.48462350C > T, and this can significantly reduce the ovine litter size in third parity (Zhang et al., 2019). There was no difference in the seven transmembrane regions between the wild type and mutated version of the MTNR1B (Figure 1). However, the result of the transmembrane regions prediction revealed that the replacement of Ile with Leu occurred outside of the cell, which may serve as a proof of this locus as a ligand‐binding site. The mutation in rs400827589 from T to G leads to the presence of a unique polynucleotide‐binding region in proximity to AA 326 position (Figure 2), and resulted in structural variation in the side chains of the tertiary structure of MTNR1B (Figure 3b). These results indicate that the change in amino acid from Ile to Leu may affect the function of the MTNR1B protein by impacting the secondary and tertiary protein structures.

As a key link in the melatonin signalling pathway, polymorphisms of *MTNR1B* gene may affect melatonin signalling by altering MTNR1B structure and expression, with changes in ovarian function (Yang et al., 2016). Ovarian development has significant effects on subsequent oestrus, ovulation and litter size. Therefore, we divided the six sheep breeds into two groups according to the oestrous characters, and found genotype frequency and allele frequency were significantly different between the two groups (*p* < .01) in SNP rs400827589 (Table 3). The results preliminarily showed that the mutation may be related to the oestrus or reproductive seasonality in ewes. Secondarily, association analysis of rs400827589 with litter sizes in Small Tail Han sheep demonstrated that ewes with TT and TG genotypes had a larger litter size than those with GG genotype. Just like the function of MTNR1A in sheep in our previous studies (Chu et al., 2006, 2008), polymorphism of *MTNR1B* in rs400827589 also affected ovine reproductive seasonality and litter size differently, with litter size of the mutant homozygote individuals decreased significantly. This phenomenon indicated that the rs400827589 may be an adverse mutation for litter size in sheep. Further study is required to confirm the mechanism of the effect of rs400827589 on the litter size in sheep.

## 5 CONCLUSIONS

The TT genotype in *MTNR1B* rs400827589 was the dominant genotype in sheep around the world. Polymorphism of *MTNR1B* in rs400827589 can affect reproductive seasonality and was associated with litter size in some Chinese native sheep. In addition, bioinformatics analysis indicated the change of amino acid from Ile to Leu may affect the function of the MTNR1B protein by impacting the secondary and tertiary protein structures. Findings in the present study also indicate that MTNR1B might use to select for litter size in sheep.

## CONFLICT OF INTEREST

We certify that there is no conflict of interest with any organization regarding the material discussed in the manuscript.

## AUTHOR CONTRIBUTION

**Xiaoyun He:** Visualization; Writing‐original draft. **Zhuangbiao Zhang:** Software. **Ming‐Xing Chu:** Formal analysis; Funding acquisition; Project administration.
